# Underlying Mechanisms of Osteoporosis in the Context of Multimorbidity: Clinical Challenges and Management Strategies

**DOI:** 10.3390/nu18020262

**Published:** 2026-01-14

**Authors:** Alberto Castagna, Carmelo Pujia, Elisa Mazza, Samantha Maurotti, Yvelise Ferro, Valeria Rizzo, Martina Formica, Rosy Conforto, Caterina Mercuri, Angela Sciacqua, Carmine Gazzaruso, Arturo Pujia, Tiziana Montalcini

**Affiliations:** 1 Department of Medical and Surgical Sciences, University “Magna Græcia” of Catanzaro, 88100 Catanzaro, Italy; a.castagna@unicz.it (A.C.); yferro@unicz.it (Y.F.); sciacqua@unicz.it (A.S.); pujia@unicz.it (A.P.); 2 O.U. Clinical Nutrition, Renato Dulbecco Hospital, 88100 Catanzaro, Italy; carmelopujia97@gmail.com (C.P.); valeria.rizzo@studenti.unicz.it (V.R.); rosy.conforto@studenti.unicz.it (R.C.); 3 Department of Clinical and Experimental Medicine, University “Magna Græcia” of Catanzaro, 88100 Catanzaro, Italy; smaurotti@unicz.it (S.M.); martina.formica@studenti.unicz.it (M.F.); c.mercuri@unicz.it (C.M.); tmontalcini@unicz.it (T.M.); 4 Research Center for the Prevention and Treatment of Metabolic Diseases, University “Magna Græcia” of Catanzaro, 88100 Catanzaro, Italy; 5 Diabetes and Endocrine and Metabolic Diseases Unit, Centre for Applied Clinical Research (Ce.R.C.A.), Clinical Institute “Beato Matteo” (Hospital Group San Donato), 27029 Vigevano, Italy; carmine.gazzaruso@unimi.it

**Keywords:** osteoporosis, comorbidity, metabolic alterations, multidisciplinary approach

## Abstract

Osteoporosis and chronic conditions such as type 2 diabetes mellitus, cardiovascular disease, heart failure, and chronic kidney disease share several common biological mechanisms, including chronic inflammation, oxidative stress, hormonal dysregulation, and metabolic alterations. In this context, multimorbidity presents an increasing clinical challenge, particularly in older populations, where osteoporosis remains frequently underdiagnosed and undertreated. This review aims to explore the complex interplay between skeletal fragility and cardiometabolic diseases, emphasizing the role of nutritional deficiencies (such as iron and vitamin C), shared molecular pathways (advanced glycation end-products, Renin–Angiotensin–Aldosterone System, RANK Ligand, RANK), and the systemic impact of chronic inflammation and tissue hypoperfusion. The review also addresses the effects of various drug classes—antidiabetics, antihypertensives, anticoagulants, and anti-osteoporotic agents—on bone metabolism and cardiovascular risk. Special focus is given to the implementation of integrated and personalized care models, particularly multidisciplinary team-based approaches, which have demonstrated significant reductions in mortality and refracture rates, despite their still limited adoption in clinical practice. In conclusion, this review highlights the shared mechanisms between osteoporosis and cardiometabolic conditions in the context of multimorbidity, underscoring persistent clinical challenges related to diagnosis, drug interactions, and care fragmentation that warrant further research into integrated care models.

## 1. Introduction

Multimorbidity is defined as the coexistence of two or more chronic conditions, without any single disease taking precedence over the others [1]. Globally, the overall prevalence of multimorbidity is estimated at 37.2%, with Europe reporting a slightly higher prevalence of 39.2% (95% CI: 33.2–45.2). Notably, multimorbidity in Europe is more common in women (43.4%) than in men (37.4%). Among individuals over the age of 60, the prevalence exceeds 51%, underscoring the clinical importance of this issue [2].

Based on WHO criteria for bone mineral density (BMD), over 32 million individuals in Europe are affected by osteoporosis, of whom more than 25 million are women [3]. Osteoporosis is a major public health concern, responsible for approximately 4.3 million fragility fractures annually across Europe [4,5].

The economic burden is substantial, with total direct costs of €56.9 billion reported in 2019. This figure includes €36.3 billion for the immediate costs of incident fractures, €19.0 billion for long-term disability care, and €1.6 billion for assessment and pharmacological treatment. Additionally, 23.8 million Europeans are estimated to have a 10-year probability of major fracture above high-risk thresholds [4].

Osteoporosis is increasingly recognized not as an isolated skeletal disease, but as part of a broader constellation of interrelated chronic conditions. These are driven by common pathophysiological mechanisms such as chronic inflammation, oxidative stress, endothelial dysfunction, and disrupted bone–vascular–metabolic interactions. Such mechanisms are shared by cardiovascular diseases (CVD), heart failure (HF), chronic kidney disease (CKD), and type 2 diabetes mellitus (T2DM) [6].

A recent population-based study in Europe showed that CKD, especially in its advanced stages, tends to cluster with CVD, osteoporosis, and hip fractures [7]. Data from real-world cohorts further indicate that over one in four individuals with T2DM may have coexisting osteoporosis [8], reinforcing the need for integrated diagnostic and therapeutic approaches.

Epidemiological evidence indicates that cardiometabolic and renal conditions are highly prevalent among individuals with osteoporosis and fragility fractures. T2DM has been reported in approximately 12.7% of patients with osteoporosis [9], reflecting a substantial burden of metabolic comorbidity in this population. CVD—including IHD and HF—is also frequently observed, with prevalence estimates reaching up to 29.4% in osteoporotic cohorts, alongside a high burden of cardiometabolic risk factors such as hypertension (up to 87.1%) and diabetes (35.6%) in combined chronic disease populations [10]. Moreover, chronic kidney disease (CKD) is closely linked to skeletal fragility: meta-analytic data indicate that approximately 24.5% of adults with CKD have low bone mineral density consistent with osteoporosis, with prevalence rising to nearly 30% among patients receiving dialysis [11]. Together, these findings highlight osteoporosis as a central component of multimorbidity, characterized by the frequent coexistence of cardiometabolic and renal disorders.

With regard to the relationship between osteoporosis and cardiovascular disease, it is important to emphasize that assessment of body composition, rather than BMI alone, is essential to understand the biological link between skeletal fragility and cardiometabolic disorders [12,13,14]. Although low BMI is a well-established risk factor for osteoporosis and high BMI is strongly associated with cardiovascular disease, this apparent divergence largely reflects the limitations of BMI as a crude anthropometric measure. BMI does not capture body composition or the distribution of muscle mass. Growing evidence indicates that reduced lean mass, particularly low skeletal muscle mass, may coexist across both low- and high-BMI categories and represents a shared determinant of osteoporosis and cardiovascular disease [15].

Current evidence highlights the urgent need to move away from disease-specific models of care and adopt a broader, mechanism-based approach, particularly in patients with multimorbidity [6]. Rather than focusing solely on individual conditions, emerging data suggest that clinical management should address the shared biological pathways that underlie coexisting chronic diseases. This patient-centered paradigm is especially relevant in older populations, where multimorbidity is highly prevalent and associated with complex healthcare needs.

The high prevalence of multimorbidity presents substantial challenges for both clinicians and patients. For healthcare professionals, it often results in fragmented and poorly coordinated care, difficulty applying disease-specific guidelines, and limited capacity to deliver truly personalized interventions. For patients, multimorbidity is associated with polypharmacy, increased treatment burden, mental health challenges, functional decline, reduced quality of life, and a heightened risk of emergency hospitalizations [16].

In this context, osteoporosis is frequently perceived as a lower-priority condition, especially when compared to more symptomatic or life-threatening illnesses such as diabetes or CVD. This perception may diminish both clinicians’ attention to bone health and patients’ willingness to adhere to osteoporosis treatments [17,18]. Furthermore, concerns regarding adverse effects or potential drug interactions with existing therapies often result in poor adherence or early discontinuation of anti-osteoporotic medications [19,20]. Structural barriers, such as limited access to parenteral therapies or Dual-energy X-ray Absorptiometry (DXA) scans, further exacerbate treatment gaps—particularly among frail, elderly, or homebound populations.

Longitudinal studies confirm that major chronic diseases in older adults are strong predictors of all-cause mortality. More importantly, the co-occurrence of multiple conditions substantially increases mortality risk [20]. It is crucial to distinguish the concept of multimorbidity from comorbidity, the latter traditionally centered around a single index disease. In contrast, multimorbidity calls for a holistic approach that prioritizes the patient’s overall clinical picture and personal goals, rather than rigid adherence to disease-specific targets.

Understanding these interrelationships in individuals with osteoporosis is essential for developing more effective and individualized treatment strategies. A truly patient-centered approach may involve recognizing that not all disease-specific guideline targets are feasible or meaningful. Instead, clinical care should aim to optimize functional status, quality of life, and health outcomes—while minimizing polypharmacy, drug–disease and drug–drug interactions, and unnecessary medical interventions.

This review aims to explore the complex interplay between osteoporosis and cardiometabolic conditions, including T2DM, CVD, HF, and CKD, within the broader framework of multimorbidity. It synthesizes current evidence on shared pathophysiological mechanisms, diagnostic challenges, therapeutic dilemmas, and the need for integrated, person-centered care models. Emphasis is placed on moving beyond fragmented, disease-specific approaches toward holistic strategies tailored to individuals with multiple coexisting chronic conditions. Such integration has the potential to reduce clinical risk, improve adherence, and enhance quality of life. In light of the limitations of conventional, single-disease guidelines in multimorbid populations, this review proposes a personalized, coordinated model of care aimed at preserving function and achieving better health outcomes across the continuum of aging.

## 2. Materials and Methods

We conducted a comprehensive narrative review of the literature to explore the relationship between osteoporosis and cardio-metabolic comorbidities in adult and older populations. Adult populations were defined as individuals aged 18 years or older. Studies exclusively involving pediatric or adolescent populations (<18 years) were excluded. This population filter was applied during the screening phase to ensure relevance to osteoporosis and multimorbidity, conditions that predominantly affect adult and older individuals. The aim of the review was to identify shared biological mechanisms underlying osteoporosis and cardiometabolic and renal diseases, as well as to summarize current clinical management strategies and models of integrated care in patients with multimorbidity. A systematic search was performed in PubMed, Embase, Scopus, and Web of Science databases for articles published from January 1995 to June 2025, restricted to studies written in English. The selected time frame reflects the period in which research on the interplay between osteoporosis and cardiovascular and metabolic diseases began to develop and consolidate, while allowing inclusion of contemporary clinical and mechanistic evidence.

Search terms included a combination of Medical Subject Headings (MeSH) and keywords related to the core themes of this review. The following terms were used with appropriate Boolean operators (AND, OR): “Osteoporosis,” “Multimorbidity,” “Comorbidity,” “Cardiovascular disease,” “Type 2 diabetes mellitus,” “Chronic kidney disease,” “Heart failure,” “Polypharmacy,” “Medication adherence,” “Patient-centered care,” “Integrated care,” “Bone-vascular axis,” “Fracture risk,” “Sarcopenia,” “Malnutrition,” “Oxidative stress,” and “Older adults.” An example of the search strategy applied in PubMed was: (“osteoporosis” OR “bone loss” OR “fragility fractures”) AND (“cardiovascular disease” OR “type 2 diabetes mellitus” OR “heart failure” OR “chronic kidney disease”) AND (“pathophysiology” OR “biological mechanisms” OR “bone-vascular axis” OR “oxidative stress”) AND (“management” OR “integrated care” OR “patient-centered care”).

Eligible sources included original research articles, systematic reviews, meta-analyses, and clinical practice guidelines that addressed osteoporosis and at least one cardio-metabolic condition in adult populations.

Studies focusing exclusively on pediatric populations, non-peer-reviewed materials, or those lacking full-text availability were excluded.

A qualitative narrative synthesis of the selected studies was conducted which refers to a structured, non-statistical approach to evidence integration, whereby findings from the included studies were systematically grouped and interpreted according to predefined thematic domains. This approach was used to identify shared biological and pathophysiological mechanisms and to synthesize clinical management strategies and models of care across heterogeneous study designs.

The data were organized thematically into six main domains: Epidemiology of multimorbidity; Epidemiology of multimorbidity and osteoporosis; Pathophysiological overlap; Pathophysiological mechanisms linking bone and cardiovascular/metabolic health; Clinical risk in the osteoporosis management in multimorbid patients; Models of integrated and personalized care.

In total, approximately 1200 records were screened across the selected databases, and approximately 95 articles were included in the qualitative synthesis. Although no formal quality scoring system was applied, included studies were appraised qualitatively based on study design, population characteristics, relevance to the review objectives, and consistency with existing evidence. When conflicting findings were identified, these were addressed narratively by contextualizing results according to differences in study populations, clinical settings, and methodological approaches.

No formal risk of bias assessment or meta-analysis was performed due to the narrative nature of the review. This methodological choice may limit reproducibility and introduce selection bias; however, it was considered appropriate given the exploratory aim of synthesizing mechanistic and clinical concepts across heterogeneous study designs.

## 3. Results

### 3.1. Osteoporosis and Diabetes

#### 3.1.1. Epidemiology

Both type 1 (T1DM) and T2DM are associated with an increased risk of fractures, although the underlying mechanisms differ between the two conditions. A 2007 meta-analysis demonstrated that while fracture risk is elevated in both forms of diabetes, BMD tends to be decreased in T1DM but is generally normal or even elevated in T2DM—likely due to higher body mass index (BMI), which is a major determinant of BMD [21]. However, the increased BMD observed in T2DM does not confer protection against fractures. This paradox may be explained by diabetes-related deterioration in bone microarchitecture—undetectable through standard BMD measurements—as well as increased fall risk, both of which contribute to greater fracture susceptibility in both T1DM and T2DM [22].

In both types of diabetes, longer disease duration has been consistently associated with a higher risk of fractures [23,24]. A recent meta-analysis reported that diabetes in general increases the risk of total fractures, with a more pronounced effect observed for hip fractures [25]. Specifically, in T1DM, the risk of any fracture is significantly increased compared to non-diabetic individuals [26], with a summary RR of approximately 3.16 (95% CI: 1.51–6.63). The risk of hip fracture is markedly elevated, with an approximately threefold increase compared with non-diabetic populations [27]. Vertebral fractures are also significantly more frequent in T1DM [26].

In T2DM, the overall risk of any fracture is moderately increased by approximately 5–24% compared to non-diabetic individuals. The risk of hip fractures rises by about 8% to 70%, with most meta-analyses reporting relative risks in the range of 1.3–1.7 [27]. The risk of non-vertebral fractures (including hip, ankle, wrist, and upper arm) is also moderately elevated, with an estimated RR of approximately 1.2 [27]. Several studies have further estimated that, in individuals with T2DM, the risk of non-vertebral fractures increases by approximately 19–20%, while the risk of hip fractures rises by around 33% [28,29].

It is well established that advanced glycation end-products (AGEs) are formed in diabetes through the non-enzymatic glycation of proteins, lipids, and nucleic acids. These compounds accumulate progressively with aging and in chronic diseases—including in bone tissue [30]. AGEs interact with specific receptors, primarily the Receptor for Advanced Glycation End-products (RAGE), triggering cascades of oxidative stress and inflammatory responses. As such, AGEs represent a crucial molecular link between osteoporosis, cardiovascular health, and diabetes. In T2DM, AGEs significantly impair bone microarchitecture, contributing to an increased risk of fractures independently of BMD. Their effects are exerted through several key mechanisms:

- Bone Collagen: Collagen plays a central role in determining the mechanical strength of bone tissue, primarily through the formation of intermolecular cross-links between adjacent collagen molecules. AGEs bind to type I collagen within the bone matrix, promoting the formation of non-enzymatic cross-links. These modifications increase collagen fiber stiffness [31].

While enzymatic cross-links enhance bone strength, non-enzymatic AGE-induced cross-linking compromises both the biological and mechanical properties of bone. As a result, bone elasticity is reduced, leading to increased fragility and a higher risk of fractures under lower mechanical loads. Interestingly, in bones with high AGE content, collagen fibrils themselves may not be the primary sites of fracture initiation, as their stiffness and strength are elevated. Instead, fractures may originate in the surrounding mineral phase, which is more brittle. This redistribution of mechanical load to the mineral component may underlie the altered fracture behavior observed in AGE-accumulated bone [31].

- Bone Cells: AGEs impair osteoblastic function through their interaction with cell-surface RAGE receptors. This interaction inhibits osteoblast activity, reducing synthesis of the bone matrix and impairing bone formation [32]. In parallel, AGEs contribute to dysregulated bone remodeling by enhancing osteoclast activity. These bone-resorbing cells may become overactivated, tipping the balance toward excessive bone resorption [33]. Osteocytes—the most abundant bone cells—are also adversely affected. AGEs induce oxidative stress in osteocytes, leading to apoptosis and disruption of the osteocyte network, ultimately impairing local bone turnover.
- Trabecular Architecture: AGE accumulation contributes to the deterioration of trabecular (spongy) bone microarchitecture, including a reduction in trabecular connectivity. This structural damage is particularly evident in clinically relevant skeletal sites such as the spine and hip. Notably, these alterations may occur even when BMD is normal or elevated—a phenomenon frequently observed in individuals with T2DM [34].
- Oxidative Stress and Inflammation: AGEs binding to RAGE also sustains a pro-oxidative and pro-inflammatory environment that further damages bone. Reactive oxygen species (ROS), in addition to causing cellular damage, act as second messengers that influence signaling pathways and gene expression. ROS enhance the activity of key apoptotic enzymes, including caspases-8, -9, and -3. In diabetes, elevated levels of tumor necrosis factor-alpha (TNF-α) exacerbate this effect, promoting apoptosis of bone cells and contributing to impaired bone remodeling and fragility [35,36].

#### 3.1.2. Common Biological Pathways: The Role of the Renin–Angiotensin–Aldosterone System (RAAS)

The RAAS plays a central role in the development of insulin resistance and structural damage in the diabetic kidney, particularly affecting intraglomerular hemodynamics and the glomerulotubular unit [37].

Angiotensin II (Ang II), the principal effector of the RAAS, exerts strong vasoconstrictive effects through activation of the angiotensin II type 1 receptor (AT1R). Importantly, RAAS components—including ACE2—are also expressed in bone tissue and bone marrow. Notably, RAAS activity is elevated in the bone marrow of individuals with T2DM [38].

In this context, the Ang II/AT1R axis promotes the release of pro-inflammatory cytokines such as interleukin-1β (IL-1β), IL-6, and TNF-α, which in turn stimulate osteoclast precursor proliferation and osteoclastogenesis. Additionally, the RANKL–RANK pathway is upregulated, further enhancing osteoclast differentiation and activity [39].

RAAS upregulation in T2DM may also increase parathyroid hormone (PTH) secretion, contributing to bone fragility [40]. Mineralocorticoid receptors (MRs), expressed in osteoblasts and osteoclasts, mediate aldosterone’s direct effects on bone. Aldosterone can worsen bone health through MR-induced PTH stimulation, hypercalciuria, and secondary hyperparathyroidism [40]. Moreover, circulating levels of renin and aldosterone have been associated with osteocalcin levels and BMD in individuals of African ancestry [41]. Supporting this, antihypertensive agents that inhibit RAAS—particularly angiotensin receptor blockers (ARBs)—have been shown in systematic reviews and meta-analyses to reduce fracture risk [42].

#### 3.1.3. Common Biological Pathways: The Role of Diabetes Medications

Observational and epidemiological studies have shed light on the potential skeletal effects of antidiabetic medications [43]. Metformin appears to reduce bone resorption and promote osteoblast activity, potentially lowering osteoporosis risk, though its precise mechanisms remain under investigation [44]. GLP-1 receptor agonists (GLP-1RAs) may promote bone formation and inhibit resorption, with some evidence supporting their benefit in postmenopausal women [45,46]. Insulin exerts dual effects, both enhancing bone formation and indirectly influencing bone metabolism via glucose regulation [43].

A weak association between SGLT2 inhibitors and fracture risk has been reported, though this may be confounded by external factors [47]. In the CANVAS Program, an increased incidence of fractures was observed in patients treated with canagliflozin versus placebo However, this effect was not replicated in the CANVAS-R trial, where pioglitazone use was minimized. This suggests the observed fracture risk in CANVAS may have been driven by concomitant pioglitazone exposure rather than canagliflozin itself [48].

**Figure 1:**
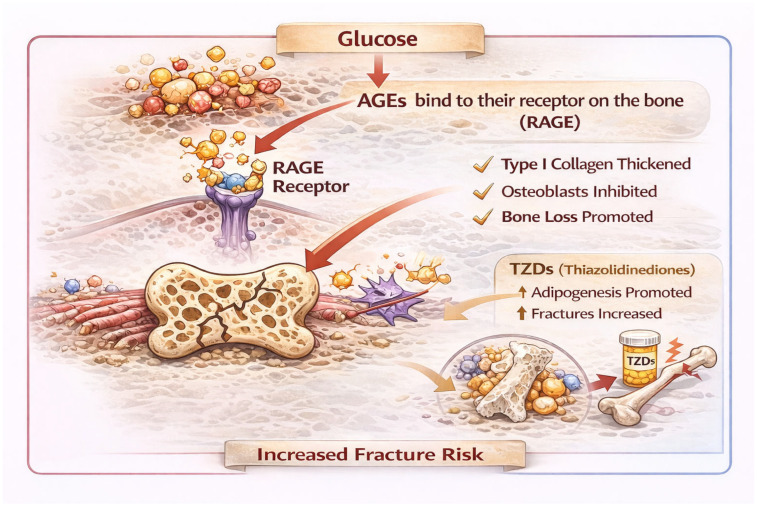
Effects of TDM and antidiabetic therapies on bone quality and fracture risk.

Thiazolidinediones (TZDs), including pioglitazone and rosiglitazone, are strongly associated with increased fracture risk, particularly in women. These agents act via PPARγ, promoting adipogenesis at the expense of osteoblastogenesis, ultimately impairing bone remodeling. Numerous in vitro studies and clinical trials confirm that TZDs induce bone loss. A recent meta-analysis found that pioglitazone significantly increases fracture risk, affecting both minor and major fractures [49]. Current clinical guidelines recommend avoiding pioglitazone, especially in postmenopausal women and men with additional risk factors for bone fragility. Current clinical guidelines recommend avoiding TZDs—especially in postmenopausal women and in men with additional risk factors for bone fragility (Figure 1).

### 3.2. Osteoporosis and Cardiovascular Disease

#### 3.2.1. Epidemiology

It is now well established that osteoporosis does not occur in isolation but often precedes, coexists with, or results from other chronic comorbidities, including cardiovascular disease (CVD) [50].

Increasing clinical evidence supports a bidirectional relationship between skeletal fragility and vascular disease. For example, prevalent vertebral fractures have been shown to predict a significantly higher risk of cardiac events in older women, while whole-body BMD loss is associated with increased cardiovascular risk in older men. Conversely, higher total hip BMD correlates with a lower incidence of HF [50,51].

A meta-analysis further confirmed this association, demonstrating a significantly elevated risk of CVD in individuals with low BMD [52].

##### Osteoporosis and Myocardial Infarction and Stroke

Major cardiovascular events such as myocardial infarction (MI) and stroke are associated with a substantially increased risk of fragility fractures. Stroke has been linked to a markedly elevated risk of hip fractures, likely due to post-event immobility, neuromuscular impairment, and increased fall risk. Similarly, myocardial infarction significantly increases hip fracture risk, and patients with a history of MI demonstrate approximately a 30% higher risk of fractures compared with individuals without prior ischemic events [50,53].

In addition, atrial fibrillation (AF)—which frequently complicates ischemic heart disease and stroke—has been associated with an increased risk of hip fractures, further supporting the close clinical link between acute cardiovascular events and skeletal fragility [50,53].

##### Osteoporosis and Heart Failure

HF has emerged as a major global public health concern, being a disabling clinical syndrome characterized by high morbidity and mortality. Importantly, HF also increases the risk of fragility fractures [54]. HF and osteoporotic fractures share several common risk factors, including aging, diabetes mellitus, smoking, menopause, and poor nutritional status [55]. A recent meta-analysis confirmed that patients with HF have a significantly higher prevalence of fractures compared to individuals without HF [56]. The analysis included a total of 260,410 participants, with individual study sample sizes ranging from 5613 to 87,748, and a median follow-up of 5.0 years. During follow-up, patients with HF had an approximately twofold increased risk of incident fractures. This elevated risk was consistent across all subgroup analyses—regardless of age, sex, sample size, or follow-up duration—with HRs ranging from 1.18 to 2.0. In particular, the risk of hip fractures was notably higher among HF patients compared to controls.

#### 3.2.2. Common Biological Pathways: The Role of Molecular Cross-Talk and Shared Signaling Mechanisms Between Bone and Cardiovascular Systems

CVD and osteoporosis are increasingly recognized as interconnected conditions that share common pathophysiological pathways. Bone and vascular tissues utilize overlapping molecular and cellular signaling mechanisms, and systemic conditions such as aging, T2DM, and chronic inflammation can contribute simultaneously to both bone loss and vascular calcification [57].

Key osteogenic proteins—including osteocalcin, osteoprotegerin (OPG), collagen, and bone morphogenetic proteins (BMPs)—are involved in both bone matrix deposition and vascular mineralization. OPG, a decoy receptor for RANKL, plays a critical regulatory role in bone remodeling by inhibiting osteoclast differentiation and activation. Interestingly, elevated circulating levels of OPG have been associated with both osteoporosis and cardiovascular disease (CVD), possibly reflecting a compensatory response to systemic inflammation or localized remodeling processes within bone and vascular tissues [58,59].

In individuals with CVD, excessive production of ROS impairs osteoblast differentiation and survival, while simultaneously promoting apoptosis and functional decline, resulting in defective bone formation [60,61]. ROS also contribute directly to vascular calcification and endothelial dysfunction, underscoring their central role in the pathophysiological link between bone and vascular systems [62].

Chronic low-grade inflammation, oxidized lipids, and endothelial dysfunction are common drivers of both vascular calcification and bone fragility, and are especially pronounced in patients with T2DM and metabolic syndrome [63,64].

A critical mechanism involves the mobilization of calcium and phosphate from bone into the circulation, which can contribute to vascular calcification under pathological conditions such as osteoporosis, chronic inflammation, oxidative stress, and renal or cardiovascular disease. In states of elevated bone resorption—such as in osteoporosis or in response to pro-resorptive stimuli like PTH and RANKL—calcium and inorganic phosphate (Pi) are released into the bloodstream. When these mineral ions accumulate and are not adequately buffered or excreted, they can precipitate in vascular tissues [65].

Furthermore, vascular smooth muscle cells (VSMCs) exposed to high levels of calcium and phosphate may undergo phenotypic transdifferentiation into osteoblast-like cells. These transformed cells begin expressing bone-related proteins such as osteocalcin, alkaline phosphatase (ALP), and BMPs [66,67]. This transformation initiates pathological vascular mineralization, mimicking physiological bone formation but in an inappropriate anatomical site. Oxidative stress further amplifies this process.

Additional pathophysiological mechanisms also support the existence of a bone–vascular axis. For example, ischemia and hypoperfusion commonly observed in CVD and HF are mirrored in bone tissue, where oxygen availability plays a key role in regulating bone cell function. Mild hypoxia induces osteoblast quiescence and enhances osteoclastogenesis via upregulation of RANKL and macrophage colony-stimulating factor (M-CSF), promoting bone resorption. Severe hypoxia (pO_2_ ≤ 2%) significantly suppresses osteoblast activity while further enhancing osteoclast function, leading to accelerated bone loss [68].

**Figure 2:**
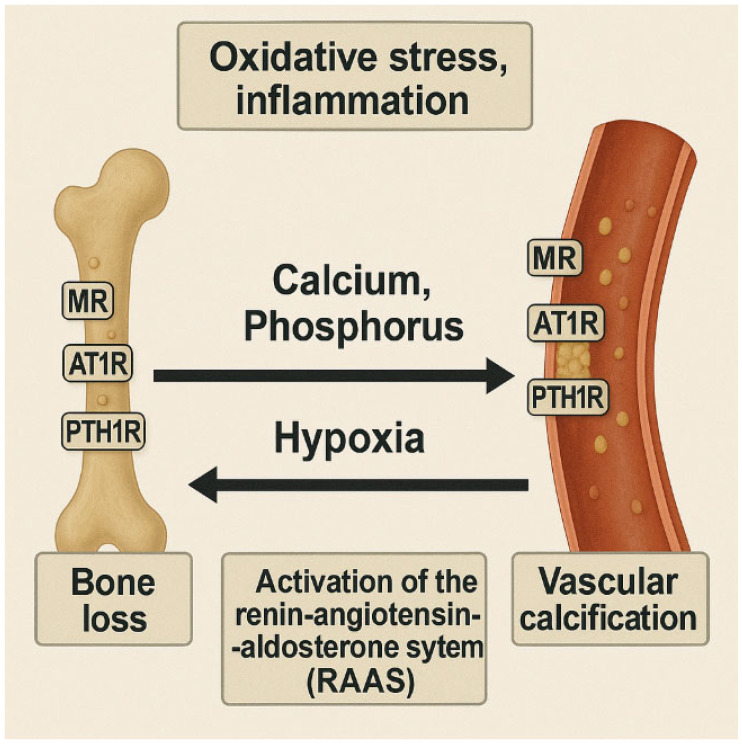
Shared molecular mechanisms between osteoporosis and vascular calcification: involvement of OPG, BMPs, and oxidative stress. Chronic inflammation, oxidative stress, and endothelial dysfunction promote bone resorption and VSMC transdifferentiation into osteoblast-like cells. RAAS activation, hypoxia, and calcium/phosphate mobilization exacerbate bone deterioration and vascular mineralization. RAAS: Renin–Angiotensin–Aldosterone System; MR: mineralocorticoid receptor; AT1R: angiotensin II type 1 receptor; PTH1R: parathyroid hormone receptor 1.

Activation of the RAAS has also been shown to exacerbate skeletal deterioration in patients with CVD and HF. This occurs through the upregulation of key receptors involved in both vascular and skeletal pathology, including the mineralocorticoid receptor (MR), AT1R, and PTH receptor 1 (PTH1R). These pathways contribute to increased bone resorption, impaired bone formation, and overall bone mass loss—further reinforcing the multifactorial and bidirectional nature of the bone–vascular axis (Figure 2) [40,69]. The main shared pathophysiological pathways are summarized in Table 1.

#### 3.2.3. Common Biological Pathways: The Role of Cardiovascular Medications in Increasing Fracture Risk

A recent large retrospective cohort study involving 29,648 long-term nursing home residents found that the initiation of antihypertensive therapy was associated with a twofold increase in fracture risk within the first 30 days of treatment (5.4 vs. 2.2 fractures per 100 person-years) [70]. However, the study lacked sufficient statistical power to assess differences in fracture risk among specific classes of antihypertensive drugs.

Antihypertensive medications may exert distinct effects on bone metabolism. Recent studies have suggested that ARBs, selective β-adrenergic receptor blockers, and thiazide diuretics may offer protective effects on bone health. These include improvements in trabecular number and BMD, likely through mechanisms such as stimulation of osteoblast differentiation and suppression of osteoclast formation [71,72,73,74].

A comprehensive review examining the relationship between hypertension and osteoporosis, with particular focus on how different classes of antihypertensives influence BMD and fracture risk, concluded that loop diuretics—such as furosemide—are associated with an increased risk of systemic fractures, especially hip fractures. This heightened risk may be attributed to their effect on increasing urinary calcium excretion and elevating circulating PTH levels, both of which contribute to systemic bone loss [71].

Additionally, a small but statistically significant increase in the risk of hip and femur fractures has been observed among users of α-adrenergic receptor blockers (α-blockers). These agents may influence bone health through vasodilation-induced changes in bone perfusion, which could impair the delivery of oxygen and nutrients necessary for bone remodeling. Moreover, α-blockers may interfere with sympathetic nervous system activity, which plays a key regulatory role in osteoblast and osteoclast function [75].

Large-scale population studies have also reported that the use of oral anticoagulants is associated with an increased risk of osteoporosis and fractures compared to non-use [76,77]. However, among patients with AF, those treated with direct oral anticoagulants (DOACs) tend to have a lower fracture risk compared to those treated with warfarin. Specifically, the initiation of apixaban or rivaroxaban has been associated with a reduced risk of any fracture compared to warfarin [75].

#### 3.2.4. Common Biological Pathways: The Role of Osteoporosis Medications in Increasing Cardiovascular Events

Bone and the cardiovascular system are so closely interconnected that medications used to treat bone loss and prevent fractures may also influence the risk of cardiovascular events. The first reports in this regard focused on bisphosphonates [78]. Notably, intravenous bisphosphonates—such as etidronate and zoledronate—have been associated with an increased risk of arrhythmias, particularly AF [79].

The first signal suggesting a link between bisphosphonates and AF emerged from major randomized controlled trials (RCTs). In the HORIZON Pivotal Fracture Trial (zoledronic acid vs. placebo), the incidence of serious AF was significantly higher in the treatment group (1.3% vs. 0.5%; *p* < 0.001) [80] A follow-up analysis of alendronate trials also showed a trend toward increased AF (1.5% vs. 1.0%; *p* = 0.07) [80].

Subsequent observational studies and meta-analyses—although yielding conflicting results—have continued to explore this association. Bisphosphonates appear to prolong the QTc interval, which may contribute to their arrhythmogenic potential [80]. Supporting this concern, pharmacovigilance data have reported 1637 cases of AF—primarily associated with zoledronate and alendronate—among 143,830 adverse drug reaction reports, making them among the most frequently implicated agents in absolute terms [81]. Despite these findings, the “2024 ESC Practical Guidelines for the Management of Atrial Fibrillation” do not specifically address antiresorptive bone agents in the context of cardiovascular risk.

However, certain osteoporosis therapies used in patients with fragility fractures may carry potential cardiovascular implications. One such agent is romosozumab, a monoclonal antibody targeting sclerostin, which was associated with a higher incidence of MI and stroke during the first year of treatment in the ARCH trial [82]. In contrast, the FRAME trial, which compared romosozumab to placebo, did not show any excess cardiovascular risk [83]. Participants in the ARCH trial were older and had a greater burden of comorbidities, which may explain the divergent findings.

A recent review [84] provided a broader assessment of the available data but did not confirm a definitive association between romosozumab and increased cardiovascular risk, emphasizing the importance of patient selection and baseline risk profiles. Nevertheless, both the manufacturer and regulatory agencies such as the European Medicines Agency (EMA) and the U.S. Food and Drug Administration (FDA) have issued warnings, stating that romosozumab is contraindicated in patients with a history of recent MI or stroke.

The Women’s Health Initiative (WHI) study evaluated the effects of hormone replacement therapy (HRT) using estrogen alone or in combination with progestins in postmenopausal women. While HRT demonstrated a significant reduction in the risk of osteoporotic fractures, including hip and vertebral fractures, it also revealed an increased risk of venous thromboembolism (VTE), particularly during the early years of treatment. This elevated thromboembolic risk represents a major limitation to the use of HRT as a first-line treatment for osteoporosis, despite its positive impact on bone health [85].

Similarly, the Heart and Estrogen/progestin Replacement Study (HERS), conducted in postmenopausal women with preexisting coronary heart disease, confirmed HRT’s beneficial effects on bone metabolism. However, it also demonstrated an increased incidence of cardiovascular and thromboembolic events, especially within the first year of therapy. As a result, HRT is not recommended for the prevention of cardiovascular disease or the treatment of osteoporosis in women with coronary artery disease [86].

These findings underline the complex risk–benefit profile of hormone replacement therapy, emphasizing the need for caution in patients with cardiovascular risk factors.

#### 3.2.5. Common Biological Pathways: The Role of Generalized and Selective Malnutrition

Malnutrition is a clinical condition frequently observed in patients with CVD, HF, CKD, and osteoporosis, and represents a significant prognostic factor across all these conditions.

A retrospective study involving over 46,000 patients with coronary artery disease found that 45.4% had mild malnutrition, while 12.1% had moderate to severe malnutrition. This condition was associated with a significantly increased risk of all-cause mortality, indicating its role as a common complication and a strong predictor of poor prognosis in this population [87]. In chronic HF, a meta-analysis reported a malnutrition prevalence of 46%, and found it to be significantly associated with increased all-cause mortality underscoring the need for accurate and routine nutritional assessment in these patients [88].

Malnutrition is also frequently observed in patients with osteoporosis, where nutritional deficiencies negatively affect bone health and increase fracture risk. Epidemiological studies have shown that a high proportion of osteoporotic individuals have insufficient intake of essential nutrients such as calcium, vitamin D, and magnesium. One study reported that 99% of participants consumed less calcium than recommended, and 99.3% had inadequate vitamin D intake—deficiencies strongly associated with reduced BMD and increased fracture risk [89]. Additionally, in elderly patients, malnutrition has been shown to increase the risk of mortality, impaired mobility, and loss of independence following hip fracture [90].

Micronutrient deficiencies—particularly of iron and vitamin C—also play a central role in both cardiovascular and bone fragility. Recent data indicate that approximately 29% of patients with acute coronary syndrome are iron deficient, a condition associated with poorer clinical outcomes [91]. Similarly, 30–50% of HF patients, including those with ischemic heart disease, experience iron deficiency, which correlates with reduced exercise capacity, lower quality of life, and higher rates of hospitalization and mortality [92].

Epidemiological studies estimate that approximately 30–50% of HF patients have iron deficiency, which may occur with or without anemia. This deficiency is associated with worsened cardiac function, reduced exercise capacity, and an overall poorer prognosis [92].

Although the prevalence of iron deficiency in osteoporotic patients is less well studied, existing evidence suggests it is common among the elderly with bone fragility. One study found that iron and other micronutrient deficiencies were frequent in this group and correlated with worsened bone health [93]. Another study in postmenopausal women with osteoporosis reported a significant prevalence of iron deficiency anemia, associated with a higher risk of fractures [94].

A study by Rizzoli et al. also highlighted that iron deficiency is common in patients with fragility fractures and may negatively affect bone healing [95].

Iron is essential for the hydroxylation of vitamin D, a critical step in its activation. This process relies on iron-dependent cytochrome enzymes that convert vitamin D into its biologically active forms. Iron deficiency disrupts this mechanism, leading to reduced levels of active vitamin D, thereby impairing calcium absorption and skeletal integrity, and contributing to the development of osteoporosis and increased fracture risk [96].

Iron also plays a vital role in collagen synthesis, a process that depends on its interaction with vitamin C. Vitamin C (ascorbic acid) is required for the activity of the enzymes prolyl hydroxylase and lysyl hydroxylase, which catalyze the hydroxylation of proline and lysine residues in procollagen—an essential step for stabilizing the collagen triple helix. These enzymes require ferrous iron (Fe^2+^) at their active sites, and vitamin C helps maintain iron in its reduced (Fe^2+^) state, thus preserving enzymatic activity. A study of 251 HF patients found that 40% had vitamin C intake below recommended levels (men < 75 mg/d, women < 60 mg/d). Those with deficiency had significantly worse cardiac event-free survival and lower quality of life at 12 months [97].

In a similar cohort of 200 HF patients, 39% were vitamin C deficient, and this deficiency was associated with elevated hs-CRP levels (>3 mg/L) and shorter cardiac event-free survival [98].

These findings underscore that vitamin C deficiency—whether measured through dietary intake or plasma levels—is common in CVD populations, including those with HF, ischemic heart disease, and post-myocardial infarction. Importantly, both iron and vitamin C are indispensable for collagen formation, a process fundamental to the structural integrity of connective tissues, including bone and blood vessels.

### 3.3. Osteoporosis and Kidney Disease

Recent studies have revealed complex mechanisms through which the kidney and bone communicate, illustrating a dynamic interplay between these two organs. Patients with impaired kidney function often exhibit a reduced capacity to regulate calcium and phosphorus metabolism, leading to disturbances in bone homeostasis [99].

In a meta-analysis, the pooled global prevalence of low BMD in patients with CKD was estimated at 24.5% (95% CI: 21.3–27.8%). Subgroup analyses revealed that prevalence estimates were similar across Europe (26%, 95% CI: 21–30%) and Asia (25%, 95% CI: 21–29%). Furthermore, dialysis patients had a significantly higher prevalence of low BMD (30%, 95% CI: 25–35%) compared to non-dialysis CKD patients (12%, 95% CI: 8–16%) [11].

The kidney maintains calcium and phosphorus balance by reabsorbing and excreting these minerals to preserve metabolic and skeletal stability. More than 98% of calcium and approximately 80% of phosphorus are reabsorbed along different segments of the renal tubule after glomerular filtration [100]. When kidney function is compromised, calcium and phosphorus regulation is disrupted. If serum levels of these minerals fall, the body compensates by mobilizing calcium and phosphorus from the bones to restore homeostasis. This adaptive mechanism leads to bone loss and an increased fracture risk [101]. In CKD, declining nephron mass reduces phosphate excretion, disrupting mineral metabolism even before overt hyperphosphatemia. FGF-23 and PTH levels rise as early as CKD stages 2–3a to maintain normal serum phosphate. Notably, the kidney is the primary organ responsible for phosphorus excretion. Erythropoietin, a nephrogenic hormone best known for its essential role in erythropoiesis, also exerts multiple effects on bone metabolism [102,103]. In addition, the synergistic effects of FGF-23 and inflammation may further complicate this vicious cycle, ultimately leading to end-organ damage and increased fracture risk [104].

These findings underscore the high burden of bone disease in CKD patients, especially among those on dialysis. Early detection and targeted management strategies should be integrated into routine clinical practice to improve bone health and reduce fracture risk in this population.

### 3.4. Osteoporosis Management in Patients with Cardiometabolic and Renal Comorbidities

#### 3.4.1. Individual with Diabetes Mellitus: Diagnosis

Patients with T2DM are at increased risk of osteoporosis and fragility fractures, even in the presence of normal or high BMD. This paradox arises from qualitative impairments in bone microarchitecture, which are not adequately captured by standard DXA measurements.

For a comprehensive assessment, the following diagnostic tools are recommended:

- DXA: To evaluate BMD.
- Bioelectrical Impedance Analysis (BIA) or DXA appendicular lean mass: To assess skeletal muscle mass, especially when low muscle mass or sarcopenia is suspected.
- FRAX^®^ Score: A widely used algorithm to estimate 10-year fracture probability based on clinical risk factors and BMD. However, in diabetic patients, FRAX tends to underestimate actual fracture risk.
- Trabecular Bone Score (TBS): Derived from lumbar spine DXA scans, TBS assesses trabecular microarchitecture and has shown particular utility in T2DM patients. It captures aspects of bone fragility not evident from BMD or FRAX alone. A TBS value < 1.23 indicates degraded microarchitecture and increased fracture risk. Multiple studies, including a recent position paper by the IOF and ESCEO, confirm that TBS predicts fracture risk independently of BMD and FRAX in T2DM [105].

#### 3.4.2. Individual with Diabetes Mellitus: Treatment

Therapeutic strategies should be tailored to the specific metabolic and pharmacologic profiles of diabetic patients. TZDs (e.g., pioglitazone, rosiglitazone) should be avoided due to their association with reduced bone formation and increased fracture risk, especially in postmenopausal women [106]. Canagliflozin (SGLT2 inhibitor) may elevate fracture risk in certain populations; while evidence is mixed, caution is advised pending further research. Bisphosphonates (e.g., alendronate, risedronate, zoledronate) remain first-line treatments for osteoporosis, with strong evidence supporting their efficacy in fracture risk reduction. Denosumab, a RANKL inhibitor, is a valid alternative or sequential therapy—particularly beneficial in patients with reduced renal function.

Calcium (1000–1200 mg/day) and vitamin D (800–1000 IU/day) supplementation is essential, particularly in elderly or institutionalized patients, where it has been shown to reduce the incidence of hip and non-vertebral fractures [107]. Adequate protein intake and resistance training are critical to support musculoskeletal health and reduce fall risk, especially in the presence of sarcopenia [108]. A multidisciplinary approach is key—incorporating nutritional support (protein and micronutrients), fall prevention strategies, individualized therapy, and promotion of long-term adherence.

#### 3.4.3. Patients with CVD: Diagnosis

Patients with atherosclerotic cardiovascular disease (ASCVD) or those at high cardiovascular risk present a clinical challenge in osteoporosis management due to overlapping risk factors and potential medication contraindications. DXA and FRAX score assessments are recommended in all patients with ASCVD or heart failure. When selecting “secondary osteoporosis” in FRAX (e.g., diabetes, glucocorticoid use), clinicians should be aware that the tool may still underestimate fracture risk in this population

#### 3.4.4. Patients with CVD: Treatment

In primary prevention (no history of fragility fractures), oral bisphosphonates are the first-line therapy unless contraindicated. Before initiating anti-osteoporotic medications, especially in secondary prevention (≥1 prior fragility fracture), a cardiovascular evaluation is recommended. This may include resting ECG, echocardiography, 24 h Holter monitoring (in cases of syncope, palpitations, suspected arrhythmia) and thromboembolic risk assessment. In secondary prevention zoledronate is considered a first-line option but should be used cautiously due to possible associations with AF [109].

Romosozumab, a sclerostin inhibitor, is effective in fracture reduction but should be avoided in patients with a history of MI or stroke, due to signals of increased cardiovascular risk [82] (see Table 2). Denosumab is a suitable option for sequential therapy in both primary and secondary prevention.

All patients should be assessed for and supplemented with iron, calcium, and vitamin D as needed—especially in those with HF, where deficiencies are common and contribute to both muscle and bone fragility.

#### 3.4.5. Patients with CKD: Diagnosis and Risk Stratification

The management of osteoporosis in patients with CKD requires a nuanced, individualized approach that accounts for the stage of renal dysfunction, underlying bone turnover status, and the presence of comorbidities. The effective management of bone loss in patients with CKD hinges on accurate diagnostic stratification, cautious selection and timing of therapy, and the integration of pharmacologic and non-pharmacologic strategies within a personalized and multidisciplinary care framework. In patients with CKD stages 1–3, the standard criteria for osteoporosis diagnosis (e.g., T-score ≤ −2.5 using DXA remain valid, and fracture risk assessment tools such as FRAX can be employed, although with some caution due to potential underestimation of risk in the presence of renal dysfunction.

In contrast, for patients with CKD stages 4–5 (eGFR < 30 mL/min/1.73 m^2^), low BMD may reflect renal osteodystrophy rather than primary osteoporosis [110]. In these cases, diagnostic interpretation must be integrated with biochemical markers of bone turnover, including PTH, serum calcium, phosphate, alkaline phosphatase, and 25-hydroxyvitamin D. Additional imaging tools such as the TBS can provide valuable insight into bone microarchitecture deterioration, particularly in patients with diabetes or those with normal BMD but high fracture risk. Although bone biopsy remains the gold standard for characterizing bone turnover in advanced CKD, it is rarely used in routine clinical practice due to its invasiveness [111].

#### 3.4.6. Patients with CKD: Therapeutic Considerations and Cautions

The therapeutic approach should be stratified based on CKD stage and bone turnover status. In CKD stages 1–3, patients may be managed similarly to the general population, including the use of antiresorptive agents such as bisphosphonates or denosumab, provided that renal function is monitored and there is no contraindication. However, in advanced CKD (stages 4–5), caution is warranted. Bisphosphonates are poorly cleared in renal impairment and may accumulate, increasing the risk of adynamic bone disease. Similarly, denosumab—although not renally excreted—can cause profound and prolonged hypocalcemia in this population and requires close biochemical monitoring. Use of vitamin D analogs (e.g., calcitriol) may improve mineral homeostasis but must be balanced against the risk of hypercalcemia and hyperphosphatemia [112].

Before initiating antiresorptive therapy in CKD stages 4–5, it is critical to establish whether the patient has high or low bone turnover. In patients with suspected adynamic bone disease (characterized by suppressed PTH and low bone formation markers), antiresorptives may be harmful and should generally be avoided. Conversely, in those with high-turnover disease and elevated fracture risk, such treatments may be appropriate under specialist supervision [113].

#### 3.4.7. Patients with CKD: Non-Pharmacologic Management

Non-pharmacologic strategies remain foundational across all CKD stages. These include optimizing calcium and vitamin D intake, nutrition support, resistance and balance training, and structured fall prevention programs. Pharmacologic regimens should always be evaluated in the context of polypharmacy and potential drug–disease or drug–drug interactions, particularly in patients with multiple comorbidities.

### 3.5. Integrated and Personalized Care for Osteoporosis in Patients with Comorbidities

Although care models specifically dedicated to osteoporosis management in the context of multimorbidity and polypharmacy are still evolving, several structured approaches have been developed that effectively integrate osteoporosis care within broader, coordinated frameworks for managing multimorbidity. Models such as Minimally Disruptive Medicine (MDM) [114], the WHO’s Integrated Care for Older People (ICOPE) [115], the ADVANTAGE Joint Action from the European Commission [116], and the HIHOPE (Holistic Integration for Healthy Longevity and Aging in Place) program [117], all promote a patient-centered, multidimensional strategy aimed at preserving intrinsic capacity and preventing functional decline. These models share key components:

- Involvement of multidisciplinary teams, including geriatricians, pharmacists, physiotherapists, nutritionists, and nurses specialized in frailty and fall prevention;
- Systematic assessment of polypharmacy and pharmacological interactions, with the goal of minimizing adverse drug events and optimizing therapeutic adherence;
- Personalized therapeutic plans tailored to the patient’s priorities, health goals, and functional reserves;
- Shared decision-making that accounts for treatment burden, autonomy, and overall quality of life.

The integrated and personalized approach to osteoporosis in patients with comorbidities may include the Fracture Liaison Service (FLS), a coordinated model for secondary fracture prevention. The FLS ensures follow-up for patients with fragility fractures, improving treatment adherence and reducing the risk of subsequent fractures [118].

Some of these models use a digital platform combined with continuous clinical monitoring to address risks related to frailty, falls, malnutrition, and polypharmacy [117]. Multidisciplinary collaboration represents a key element of these models: Osteoporosis management involves not only endocrinologists, orthopedics or rheumatologists but also cardiologists, diabetologists, geriatricians, physiotherapists, and clinical pharmacists. For example, when treating a patient with T2DM, careful selection of anti-osteoporotic agents must be coordinated with the diabetes care team to avoid agents like TZDs or SGLT2 inhibitors that may exacerbate fracture risk. Similarly, in patients with CVD, cardiologists must be engaged when considering drugs like zoledronate or romosozumab due to their potential arrhythmogenic or pro-thrombotic effects.

Frailty-related factors frequently mediate the association between osteoporosis and multimorbidity. In particular, reduced muscle mass and impaired physical performance may increase fall risk and worsen functional outcomes after fragility fractures, thereby amplifying skeletal vulnerability in older adults with cardiometabolic and renal comorbidities. Even in the absence of severely reduced bone mineral density, impaired muscle function represents a relevant contributor to fracture risk and loss of independence. For this reason, integrated care pathways should routinely include the assessment of physical performance and nutritional status alongside bone-specific evaluation [108,119,120].

Dietitians and physiotherapists play a crucial role in managing sarcopenia, maintaining muscle mass, and preventing falls. Adequate intake of calcium, vitamin D, protein, and essential micronutrients are, thus, ensured, particularly in frail or elderly individuals. Exercise regimens tailored to the patient’s functional capacity—including resistance training—should be integrated into routine care to optimize musculoskeletal resilience [121].

This integrated approach is critical in ensuring that osteoporosis does not remain an overlooked comorbidity in the context of complex patient profiles. It also highlights the need to move beyond disease-specific protocols toward flexible, patient-focused strategies that optimize both bone health and overall outcomes in older adults.

### 3.6. Perspective and Future Research

#### 3.6.1. Telemedicine and Digital Health in Managing Osteoporosis in Multimorbidity

The future of osteoporosis care—particularly in patients with multimorbidity—may lie in the adoption of telemedicine and digital health technologies. These tools offer a transformative opportunity to shift from reactive, episodic care to proactive, continuous, and personalized management, especially for older adults with multiple chronic conditions. Digital platforms can facilitate remote monitoring of bone health parameters, medication adherence, fall risk, nutrition, and physical activity, while integrating data across comorbidities. Importantly, telehealth enables multidisciplinary coordination, allowing specialists such as endocrinologists, geriatricians, cardiologists, and pharmacists to collaboratively manage complex cases without geographical or logistical barriers.

In the context of osteoporosis, such technologies can be harnessed to deliver remote fracture risk assessments, monitor adherence to antiresorptive therapies, and integrate alerts for drug–disease and drug–drug interactions [122,123]. As population aging accelerates and healthcare systems face increasing pressure, digital health offers a scalable, efficient, and patient-centered solution to ensure osteoporosis is no longer neglected within the broader multimorbidity landscape.

To improve the efficiency of osteoporosis care, eConsults allow bone health specialists to remotely support primary care providers. One study showed that rheumatology eConsults significantly reduced wait times and enabled 40% of referrals to be managed without in-person visits [124]. In post-fragility fracture care, eConsult implementation led to increased prescriptions of bisphosphonates and calcium/vitamin D for secondary fracture prevention [125,126].

#### 3.6.2. Osteoporosis and CVD: A Shared Future Perspective

The coexistence of osteoporosis and CVD represents a unique clinical paradigm characterized by a bidirectional and clinically relevant interaction. Cardiovascular disease increases fracture risk through shared mechanisms such as inflammation, oxidative stress, vascular dysfunction, malnutrition, and physical deconditioning, while osteoporotic fractures—particularly hip fractures—are associated with increased cardiovascular events and mortality [127,128,129]. Future research should move beyond simple co-occurrence models and focus on defining causal pathways, temporal relationships, and high-risk phenotypes within the bone–cardiovascular axis.

A key priority is the systematic evaluation of pharmacological trade-offs. Widely used cardiovascular therapies, including loop diuretics and some antihypertensive agents, may adversely affect bone health through increased urinary calcium loss or impaired mineral metabolism [130,131]. Conversely, certain osteoporosis treatments require careful cardiovascular risk stratification. Prospective studies and real-world analyses are needed to quantify these risks and to develop mitigation strategies.

From a clinical perspective, greater emphasis should be placed on interdisciplinary training addressing bone–cardiovascular drug interactions and integrated risk assessment [132]. Examples of scalable approaches already exist, including a structured mentorship/training programs to implement effective Fracture Liaison Services, supporting the development of dedicated bone specialists and integrated models of care [132].

Current clinical guidelines largely address osteoporosis and cardiovascular disease in isolation; future updates should explicitly incorporate cardiovascular considerations into osteoporosis management and vice versa. Finally, the development of decision-support tools integrating fracture risk, cardiovascular risk [133], nutritional status, and pharmacological profiles may enable truly personalized treatment strategies, optimizing skeletal outcomes while minimizing cardiovascular harm in multimorbid patients.

In patients with coexisting osteoporosis and cardiovascular disease, digital health tools may enable integrated monitoring of fracture risk and cardiovascular status, particularly after myocardial infarction or stroke. Such platforms could support continuity and safety of anti-osteoporotic care by integrating longitudinal bone risk assessment with cardiovascular follow-up and automated alerts for falls, mobility decline, or drug–disease interactions in high-risk patients.

#### 3.6.3. Comprehensive Risk Assessment

Beyond standard BMD testing with DXA, tools such as the TBS should be routinely employed—especially in patients with diabetes—to detect microarchitectural deterioration that BMD alone cannot capture [105].

Cardiovascular risk should also be formally assessed before initiating medications with potential vascular implications, integrating resting ECG, echocardiography, or thromboembolic risk screening as needed. A clinical pharmacist or pharmacologist should systematically review all medications to identify potential adverse interactions between osteoporosis therapies and treatments for diabetes or CVD. Treatment plans should reflect the patient’s preferences, life expectancy, functional goals, and tolerance for risk. This may mean prioritizing fall prevention and quality of life over aggressive pharmacologic targets in certain populations, especially in those with limited mobility, cognitive decline, or multiple competing health priorities.

## 4. Conclusions

Patients with chronic conditions such as diabetes mellitus, CVD, CKD, and HF face a markedly elevated risk of osteoporosis and fractures. This vulnerability is driven by complex, multifactorial mechanisms including impaired bone metabolism, polypharmacy, nutritional deficiencies, reduced physical function, and increased fall risk. To address this challenge, osteoporosis care in multimorbid populations must evolve beyond standard protocols. An effective and comprehensive management strategy for osteoporosis must be integrated and personalized. It should incorporate advanced diagnostic tools—such as DXA, FRAX, and TBS—to assess bone fragility beyond BMD, particularly in high-risk populations like patients with diabetes. Prior to initiating antiresorptive or anabolic therapies, cardiovascular risk should be evaluated, and a thorough pharmacologic review conducted to identify potential drug–disease and drug–drug interactions across coexisting treatments. Treatment plans should be tailored to each patient’s functional status, life expectancy, risk tolerance, and personal preferences, with a strong focus on fall prevention and maintaining quality of life—especially in cases where aggressive therapy may not be appropriate.

Moreover, the adoption of telemedicine and digital health tools should be strongly encouraged as they offer scalable solutions to enhance care delivery in osteoporosis. These technologies can facilitate remote monitoring of fracture risk, treatment adherence, mobility, and lifestyle factors, while also reducing access barriers and wait times, and improving medication uptake for secondary fracture prevention. Implementing proactive, patient-centered strategies is essential to ensure that osteoporosis receives appropriate attention within the broader framework of chronic disease management. By embracing this integrated model of care, healthcare systems can enhance clinical outcomes, improve patient well-being, and use healthcare resources more efficiently.

## Figures and Tables

**Table 1:** Shared pathophysiological mechanisms linking osteoporosis and cardiovascular disease.

Shared Pathway	Effects on Bone	Effects on Cardiovascular System	Key Mediators	References
Chronic inflammation	Increased bone resorption, impaired formation	Endothelial dysfunction, atherosclerosis	TNF-α, IL-6, IL-1β	[57,58,63,64]
Oxidative stress	Osteoblast apoptosis, microarchitectural damage	Vascular calcification, endothelial damage	ROS	[60,61]
Bone–vascular axis dysregulation	Altered bone remodeling	VSMC osteogenic transdifferentiation	OPG, BMPs, osteocalcin	[57,58,66,67]
Mineral metabolism imbalance	Bone demineralization	Pathological vascular calcification	Calcium, phosphate, PTH, FGF-23	[66,67]
RAAS activation	Increased osteoclastogenesis	Hypertension, cardiac remodeling	Ang II, aldosterone, MR	[40,69]
Hypoxia	Reduced osteoblast activity	Ischemic damage	HIF-related pathways	[68]
AGE accumulation	Reduced bone quality	Vascular stiffness	AGE–RAGE interaction	[30]

AGE, advanced glycation end-products; BMPs, bone morphogenetic proteins; FGF-23, fibroblast growth factor-23; HIF, hypoxia-inducible factor; IL, interleukin; MR, mineralocorticoid receptor; OPG, osteoprotegerin; PTH, parathyroid hormone; RAAS, renin–angiotensin–aldosterone system; RAGE, receptor for advanced glycation end-products; ROS, reactive oxygen species; TNF-α, tumor necrosis factor-alpha; VSMC, vascular smooth muscle cell.

**Table 2:** Pharmacological Treatments for Osteoporosis in Patients with Diabetes and Cardiovascular Disease.

Drug	Risks	Clinical Practice	References
Thiazolidinediones	Bone loss, ↑ fracture risk	Avoid in osteoporotic patients	[106]
Glifozine	Fracture risk? ↑	Caution in osteoporotic patients	[48]
Zoledronate	Possible ↑ risk of AF	Use with caution in patients with AF or arrhythmias	[78,79,80,109]
Romosozumab	↑ risk of MI and stroke	Avoid in recent ischemic event or high CV risk	[82,83,84]

AF: Atrial Fibrillation; MI: Myocardial Infarction; CV: Cardiovascular.

## Data Availability

No new data were created or analyzed in this study.

## Abbreviations

The following abbreviations are used in this manuscript:

ACE2	Angiotensin-Converting Enzyme 2
AF	Atrial Fibrillation
AGEs	Advanced Glycation End-Products
ALP	Alkaline Phosphatase
ARBs	Angiotensin Receptor Blockers
ASCVD	Atherosclerotic Cardiovascular Disease
AT1R	Angiotensin II Type 1 Receptor
BIA	Bioelectrical Impedance Analysis
BMI	Body Mass Index
BMD	Bone Mineral Density
BMPs	Bone Morphogenetic Proteins
CHF	Chronic Heart Failure
CKD	Chronic Kidney Disease
CV	Cardiovascular
CVD	Cardiovascular Disease
DXA	Dual-Energy X-ray Absorptiometry
ECG	Electrocardiogram
eGFR	Estimated Glomerular Filtration Rate
EMA	European Medicines Agency
FDA	U.S. Food and Drug Administration
FGF-23	Fibroblast Growth Factor 23
FLS	Fracture Liaison Service
FRAX	Fracture Risk Assessment Tool
GLP-1RAs	Glucagon-Like Peptide-1 Receptor Agonists
HF	Heart Failure
HERS	Heart and Estrogen/progestin Replacement Study
HRT	Hormone Replacement Therapy
hs-CRP	High-sensitivity C-Reactive Protein
ICOPE	Integrated Care for Older People
IOF	International Osteoporosis Foundation
IL-1β	Interleukin-1 beta
IL-6	Interleukin-6
M-CSF	Macrophage Colony-Stimulating Factor
MI	Myocardial Infarction
MR	Mineralocorticoid Receptor
OPG	Osteoprotegerin
PPARγ	Peroxisome Proliferator-Activated Receptor gamma
PTH	Parathyroid Hormone
PTH1R	Parathyroid Hormone Receptor 1
RAAS	Renin–Angiotensin–Aldosterone System
RAGE	Receptor for Advanced Glycation End-Products
RANK	Receptor Activator of Nuclear Factor κB
RANKL	Receptor Activator of Nuclear Factor κB Ligand
RCTs	Randomized Controlled Trials
ROS	Reactive Oxygen Species
SGLT2	Sodium–Glucose Cotransporter 2
T1DM	Type 1 Diabetes Mellitus
T2DM	Type 2 Diabetes Mellitus
TBS	Trabecular Bone Score
TZDs	Thiazolidinediones
VSMCs	Vascular Smooth Muscle Cells
VTE	Venous Thromboembolism
WHO	World Health Organization
